# Supplementary material for: Perceptions and experiences of older adults in a youth community health volunteer–led health coaching program in Singapore: A qualitative study
Source: PLoS One. 2025 Nov 14;20(11):e0335716. doi: 10.1371/journal.pone.0335716 (PMC12617845; doi:10.1371/journal.pone.0335716)
Supplement: S1 Table — (DOCX) [file pone.0335716.s001.docx]

# S1 Table. Interview Guide for Older Adult Participants

| **Domain** | **Main Question** | **Probes** |
| --- | --- | --- |
| **Participant Background** | 1. Which estate do you live in? 2. When did you attend the screening program organized by Singapore General Hospital? | – |
| **Perceptions of Program & Outcomes** | 1. What was your experience going through the program? | – |
|  | 2. How did you follow up with a GP after the program? | - If yes, what led you to follow up? - If no, what were the barriers? - How was this decision influenced more by volunteers? |
|  | 3. Can you share about the healthy lifestyle goals you set? (e.g., diet, exercise) | - Were you able to achieve/maintain them? - Why or why not? - How were these influenced by volunteers or the screening? |
|  | 4. How did you understand your screening results? | - How serious did you think they were? - Were any results new to you? - Which aspects of the program helped? |
|  | 5. Have you used digital health apps (e.g., HealthHub, Healthy365, Singpass), and did this change after the program? | - If yes, what supported or hindered your use? - Were changes influenced by volunteers or the screening? |
|  | 6. How confident are you to take steps to care for your health? | - Has your confidence changed after the program? - Was this influenced by volunteers or the screening? - How confident are you to continue these behaviors without follow-up? |
| **Perceptions of YCHVs** | 7. How was your experience interacting with the YCHVs? | - How effective/helpful were they? - What was your favorite and least favorite part? - How could the experience be improved? - What are your thoughts on having youth volunteers in this role? |
| **Program Recommendations** | 8. What did you like most about the program? 9. How could it be improved? | - Suggestions to support doctor follow-up? - Suggestions to support healthy lifestyle choices? |
| **Closing** | 10. Is there anything else you would like to share that we have not covered? | – |
