# Supplementary material for: Perceptions and experiences of older adults in a youth community health volunteer–led health coaching program in Singapore: A qualitative study
Source: PLoS One. 2025 Nov 14;20(11):e0335716. doi: 10.1371/journal.pone.0335716 (PMC12617845; doi:10.1371/journal.pone.0335716)
Supplement: S2 Table — (DOCX) [file pone.0335716.s002.docx]

**S2 Table. COREQ (COnsolidated criteria for REporting Qualitative Research) Checklist**

| **Topic** | **Item No.** | **Guided Questions/Descriptions** | **Reported on Page No.** |
| --- | --- | --- | --- |
| **Domain 1: Research team and reflexivity** | | | |
| Personal characteristics | | | |
| Interview/facilitator | 1 | Which author/s conducted the interview or focus group? | Page 5: Four trained co-authors (ASTK, JYRC, JJ, EWS) conducted interviews. |
| Credentials | 2 | What were the researcher’s credentials? E.g., PhD, MD | Page 6: Public health researchers/researchers-in-training; degrees not specifically listed |
| Occupation | 3 | What was their occupation at the time of the study? | Page 5-6: Affiliated with PHIC, Singapore General Hospital; roles as researchers stated. |
| Gender | 4 | Was the researcher male or female? | Not reported |
| Experience and training | 5 | What experience or training did the researcher have? | Page 5: Relatively new to qualitative research; trained in the interview guide; supervised by qualitative lead. |
| Relationship with participants | | | |
| Participant established | 6 | Was a relationship established prior to study commencement? | Page 6: Minimal prior contact with participants before the study. |
| Participant knowledge of the interviewer | 7 | What did the participants know about the researcher? E.g., personal goals, reasons for doing the research | Page 6: Participants were informed that interviewers were researchers from SGH and TriGen evaluating the HealthStart program. |
| Interviewer characteristics | 8 | What characteristics were reported about the interviewer/facilitator? E.g., Bias assumption, reasons and interests in the research topic | Page 6: Positionality acknowledged; potential influence and mitigation described. |
| **Domain 2: Study design** | | | |
| Theoretical framework | | | |
| Methodological orientation and Theory | 9 | What methodological orientation was stated to underpin the study? E.g., grounded theory, discourse analysis, ethnography, phenomenology, content analysis | Page 6: Thematic analysis (Braun & Clarke). |
| Participant selection | | | |
| Sampling | 10 | How were the participants selected? E.g., purposive convenience, consecutive, snowball | Page 5: Heterogeneous purposive sampling. |
| Method of approach | 11 | How were the participants approached? E.g., face-to-face, telephone, mail, email | Page 3: Eligible participants were approached by phone or text messages after screening and invited to participate. |
| Sample size | 12 | How many participants were in the study? | Page 5,7: N = 19. |
| Non-participation | 13 | How many people refused to participate or dropped out? | Page 3-4: A few individuals declined participation, though the exact number was not systematically recorded. |
| Setting | | | |
| Setting of data collection | 14 | Where was the data collected? E.g., home, clinic, workplace | Page 5: Quiet rooms in community centers/healthcare facilities; or by telephone/Zoom. |
| Presence of non-participants | 15 | Was anyone else present besides the participants and researchers? | Page 5: Only the participant and interviewer were present during interviews. |
| Description of sample | 16 | What are the important characteristics of the sample? E.g., demographic, data, date | Page 7: Table 1 provides demographics and YCHV engagement; follow-up status included. |
| Data collection | | | |
| Interview guide | 17 | Were questions, prompts, guides provided by the authors? Was it pilot tested? | Page 4: Guide developed from Theory of Change; provided as S1 Table. |
| Repeat interviews | 18 | Were repeat interviews carried out? If yes, how many? | Not reported |
| Audio/visual recording | 19 | Did the research use audio or visual recording to collect the data? | Page 6: Audio-recorded; transcription checked for verbatim accuracy; identifiers redacted. |
| Field notes | 20 | Were field notes made during and/or after the interview or focus group? | Page 5: Each interview lasted approximately 60 minutes, was scheduled at participants’ convenience, audio-recorded, and accompanied by field notes documenting contextual observations. |
| Duration | 21 | What was the duration of the interviews or focus group? | Page 5: Approximately 60 minutes per interview. |
| Data saturation | 22 | Was data saturation discussed? | Page 4: Saturation determined collectively when no new insights emerged. |
| Transcripts returned | 23 | Were transcripts returned to participants for comment and/or correction? | Page 6: Transcripts were not returned to participants for comment or correction. |
| **Domain 3: Analysis and findings** | | | |
| Findings | | | |
| Number of data coders | 24 | How many data coders coded the data? | Page 6: Four coders; discrepancies resolved through discussion. |
| Description of the coding tree | 25 | Did authors provide a description of the coding tree? | Page 7-20: Themes and sub-themes described |
| Derivation of themes | 26 | Were themes identified in advance or derived from the data? | Page 6: Data was analyzed thematically using deductive and inductive approach; iterative theme development. |
| Software | 27 | What software, if applicable, was used to manage the data? | Page 6: QSR NVivo 14 was used for data management and analysis. |
| Participant checking | 28 | Did participants provide feedback on the findings? | Not reported |
| Reporting | | | |
| Quotations presented | 29 | Were participants quotations presented to illustrate the themes/findings? Was each quotation identified? E.g., participant number? | Page 7-20: Verbatim participant quotations with IDs (OAXX/ RXX) provided. |
| Data and findings consistent | 30 | Was there consistency between the data presented and the findings? | Page 7-20: Findings were supported by multiple quotations and narrative synthesis. |
| Clarity of major themes | 31 | Were major themes clearly presented in the findings? | Page 7-20: Five major themes were clearly outlined with sub-themes. |
| Clarity of minor themes | 32 | Is there a description of diverse cases or discussion of minor themes? | Page 10,14,15: Minor and divergent themes were identified (e.g., preference for Traditional Chinese Medicine, viewing conditions as mild/not requiring follow-up, and concerns about food costs). These were described in the Results section alongside major themes, though not always labeled as separate sub-themes. |

Developed from: Tong A, Sainsbury P, Craig J. Consolidated criteria for reporting qualitative research (COREQ): a 32-item checklist for interviews and focus groups. International Journal for Quality in Health Care. 2007. Volume 19, Number 6: pp. 349 – 357
