# Supplementary material for: Perceptions and experiences of older adults in a youth community health volunteer–led health coaching program in Singapore: A qualitative study
Source: PLoS One. 2025 Nov 14;20(11):e0335716. doi: 10.1371/journal.pone.0335716 (PMC12617845; doi:10.1371/journal.pone.0335716)
Supplement: Attachment — Submitted filename: Response to Reviewers.docx [file pone.0335716.s004.docx]

PONE-D-25-28987

What do Older Adults Think of Youth Community Health Volunteers (YCHVs)? Perceptions and Experiences of Older Adults in a Youth-Led Post-Health Screening Advocacy Program in Singapore: A Qualitative Study

PLOS ONE

Dear Dr. Ng,

Thank you for submitting your manuscript to PLOS ONE. After careful consideration, we feel that it has merit but does not fully meet PLOS ONE’s publication criteria as it currently stands. Therefore, we invite you to submit a revised version of the manuscript that addresses the points raised during the review process.

Please submit your revised manuscript by Oct 03 2025 11:59PM. If you will need more time than this to complete your revisions, please reply to this message or contact the journal office at plosone@plos.org. When you're ready to submit your revision, log on to [https://ddei5-0-ctp.trendmicro.com:443/wis/clicktime/v1/query?url=https%3a%2f%2fwww.editorialmanager.com%2fpone%2f&umid=FDD5C04E-3CB4-8206-8CB9-F3816F9BE5A2&auth=506d7fb60e41bcf4ca9a42eb3a951ef6efc5338b-fe97e3a4a581ef3a7e7a3286d3b6c510db5a8fc4](https://ddei5-0-ctp.trendmicro.com/wis/clicktime/v1/query?url=https%3a%2f%2fwww.editorialmanager.com%2fpone%2f&umid=FDD5C04E-3CB4-8206-8CB9-F3816F9BE5A2&auth=506d7fb60e41bcf4ca9a42eb3a951ef6efc5338b-fe97e3a4a581ef3a7e7a3286d3b6c510db5a8fc4) and select the 'Submissions Needing Revision' folder to locate your manuscript file.

Please include the following items when submitting your revised manuscript:

- A rebuttal letter that responds to each point raised by the academic editor and reviewer(s). You should upload this letter as a separate file labeled 'Response to Reviewers'.
- A marked-up copy of your manuscript that highlights changes made to the original version. You should upload this as a separate file labeled 'Revised Manuscript with Track Changes'.
- An unmarked version of your revised paper without tracked changes. You should upload this as a separate file labeled 'Manuscript'.

If you would like to make changes to your financial disclosure, please include your updated statement in your cover letter. Guidelines for resubmitting your figure files are available below the reviewer comments at the end of this letter.

If applicable, we recommend that you deposit your laboratory protocols in [protocols.io](http://protocols.io/) to enhance the reproducibility of your results. Protocols.io assigns your protocol its own identifier (DOI) so that it can be cited independently in the future. For instructions see: [https://ddei5-0-ctp.trendmicro.com:443/wis/clicktime/v1/query?url=https%3a%2f%2fjournals.plos.org%2fplosone%2fs%2fsubmission%2dguidelines%23loc%2dlaboratory%2dprotocols&umid=FDD5C04E-3CB4-8206-8CB9-F3816F9BE5A2&auth=506d7fb60e41bcf4ca9a42eb3a951ef6efc5338b-996e3e80a4d1f71787c465f647d541008d7f45ed](https://ddei5-0-ctp.trendmicro.com/wis/clicktime/v1/query?url=https%3a%2f%2ftrack.editorialmanager.com%2fCL0%2fhttps%3a%252F%252Fjournals.plos.org%252Fplosone%252Fs%252Fsubmission%2dguidelines%2523loc%2dlaboratory%2dprotocols%2f1%2f010f0198c1aa9ae5%2d72b763b3%2dae12%2d4657%2db757%2d5efd13e8916e%2d000000%2fVHY%5fQci9P2gpm00axnB02a6BNUV2mVCSSfDAmQZdWk0%3d224&umid=FDD5C04E-3CB4-8206-8CB9-F3816F9BE5A2&auth=506d7fb60e41bcf4ca9a42eb3a951ef6efc5338b-f0ce37c5434bf1b5b2c8868dcaa67e6b5f926b4e). Additionally, PLOS ONE offers an option for publishing peer-reviewed Lab Protocol articles, which describe protocols hosted on [protocols.io](http://protocols.io/). Read more information on sharing protocols at [https://ddei5-0-ctp.trendmicro.com:443/wis/clicktime/v1/query?url=https%3a%2f%2fplos.org%2fprotocols%3futm%5fmedium%3deditorial%2demail%26utm%5fsource%3dauthorletters%26utm%5fcampaign%3dprotocols&umid=FDD5C04E-3CB4-8206-8CB9-F3816F9BE5A2&auth=506d7fb60e41bcf4ca9a42eb3a951ef6efc5338b-56c50b6d350775d0017acea9b247a68ca0f87807](https://ddei5-0-ctp.trendmicro.com/wis/clicktime/v1/query?url=https%3a%2f%2ftrack.editorialmanager.com%2fCL0%2fhttps%3a%252F%252Fplos.org%252Fprotocols%253Futm%5fmedium%3deditorial%2demail%2526utm%5fsource%3dauthorletters%2526utm%5fcampaign%3dprotocols%2f1%2f010f0198c1aa9ae5%2d72b763b3%2dae12%2d4657%2db757%2d5efd13e8916e%2d000000%2fVSEjEeOsBLczl9W59zac3hAG2Mi3iZEtNByeCToeBI8%3d224&umid=FDD5C04E-3CB4-8206-8CB9-F3816F9BE5A2&auth=506d7fb60e41bcf4ca9a42eb3a951ef6efc5338b-72fc2a12f1aa1aa8072655f1ea71a70fd439f65a).

We look forward to receiving your revised manuscript.

Kind regards,

Morufu Olalekan Raimi, Ph.D
Academic Editor
PLOS ONE

Journal Requirements:

When submitting your revision, we need you to address these additional requirements.

1. Please ensure that your manuscript meets PLOS ONE's style requirements, including those for file naming. The PLOS ONE style templates can be found at

[https://ddei5-0-ctp.trendmicro.com:443/wis/clicktime/v1/query?url=https%3a%2f%2fjournals.plos.org%2fplosone%2fs%2ffile%3fid%3dwjVg%2fPLOSOne%5fformatting%5fsample%5fmain%5fbody.pdf&umid=FDD5C04E-3CB4-8206-8CB9-F3816F9BE5A2&auth=506d7fb60e41bcf4ca9a42eb3a951ef6efc5338b-ca4814fbbe46b72b0e0758247bfc4383b9888593](https://ddei5-0-ctp.trendmicro.com/wis/clicktime/v1/query?url=https%3a%2f%2fjournals.plos.org%2fplosone%2fs%2ffile%3fid%3dwjVg%2fPLOSOne%5fformatting%5fsample%5fmain%5fbody.pdf&umid=FDD5C04E-3CB4-8206-8CB9-F3816F9BE5A2&auth=506d7fb60e41bcf4ca9a42eb3a951ef6efc5338b-ca4814fbbe46b72b0e0758247bfc4383b9888593) and

[https://ddei5-0-ctp.trendmicro.com:443/wis/clicktime/v1/query?url=https%3a%2f%2fjournals.plos.org%2fplosone%2fs%2ffile%3fid%3dba62%2fPLOSOne%5fformatting%5fsample%5ftitle%5fauthors%5faffiliations.pdf&umid=FDD5C04E-3CB4-8206-8CB9-F3816F9BE5A2&auth=506d7fb60e41bcf4ca9a42eb3a951ef6efc5338b-8a16b144b2bf8010dc49261381348b0fd0789156](https://ddei5-0-ctp.trendmicro.com/wis/clicktime/v1/query?url=https%3a%2f%2ftrack.editorialmanager.com%2fCL0%2fhttps%3a%252F%252Fjournals.plos.org%252Fplosone%252Fs%252Ffile%253Fid%3dba62%252FPLOSOne%5fformatting%5fsample%5ftitle%5fauthors%5faffiliations.pdf%2f1%2f010f0198c1aa9ae5%2d72b763b3%2dae12%2d4657%2db757%2d5efd13e8916e%2d000000%2fFQtzO5mqodOgG8Z59cN%5f65yPzItSMwwasyc58a%2d0Q0Y%3d224&umid=FDD5C04E-3CB4-8206-8CB9-F3816F9BE5A2&auth=506d7fb60e41bcf4ca9a42eb3a951ef6efc5338b-568ffb5fc042155c290b15b6ec2806c716311785)

**Response**: We have reviewed the manuscript and Supporting Information files to ensure compliance with PLOS ONE style requirements, including file naming conventions. File names and formatting have been updated accordingly in line with the provided guidelines.

2. Thank you for stating the following financial disclosure:
This study is supported by the Singapore Ministry of Health’s National Medical Research Council under the Fellowship Programme by SingHealth Regional Health System, Population-based, Unified, Learning System for Enhanced and Sustainable (PULSES) Health Centre Grant (NMRC/CG/C027/2017_SHS), the Healthy, Empowered and Active Living (HEAL) fund and the Infocomm Media Development Authority Digital for Life Fund.

Please state what role the funders took in the study. If the funders had no role, please state: "The funders had no role in study design, data collection and analysis, decision to publish, or preparation of the manuscript."
If this statement is not correct you must amend it as needed.
Please include this amended Role of Funder statement in your cover letter; we will change the online submission form on your behalf.

**Response**: Thank you for your comment. The funders had no role and we have included the line "The funders had no role in study design, data collection and analysis, decision to publish, or preparation of the manuscript." in the updated cover letter as attached.

3. In this instance it seems there may be acceptable restrictions in place that prevent the public sharing of your minimal data. However, in line with our goal of ensuring long-term data availability to all interested researchers, PLOS’ Data Policy states that authors cannot be the sole named individuals responsible for ensuring data access ([https://ddei5-0-ctp.trendmicro.com:443/wis/clicktime/v1/query?url=http%3a%2f%2fjournals.plos.org%2fplosone%2fs%2fdata%2davailability%23loc%2dacceptable%2ddata%2dsharing%2dmethods&umid=FDD5C04E-3CB4-8206-8CB9-F3816F9BE5A2&auth=506d7fb60e41bcf4ca9a42eb3a951ef6efc5338b-1490ffc7a6f48f6fc71babbd5809e34f4e78fd1a](https://ddei5-0-ctp.trendmicro.com/wis/clicktime/v1/query?url=http%3a%2f%2fjournals.plos.org%2fplosone%2fs%2fdata%2davailability%23loc%2dacceptable%2ddata%2dsharing%2dmethods&umid=FDD5C04E-3CB4-8206-8CB9-F3816F9BE5A2&auth=506d7fb60e41bcf4ca9a42eb3a951ef6efc5338b-1490ffc7a6f48f6fc71babbd5809e34f4e78fd1a)).

Data requests to a non-author institutional point of contact, such as a data access or ethics committee, helps guarantee long term stability and availability of data. Providing interested researchers with a durable point of contact ensures data will be accessible even if an author changes email addresses, institutions, or becomes unavailable to answer requests.

Before we proceed with your manuscript, please also provide non-author contact information (phone/email/hyperlink) for a data access committee, ethics committee, or other institutional body to which data requests may be sent. If no institutional body is available to respond to requests for your minimal data, please consider if there any institutional representatives who did not collaborate in the study, and are not listed as authors on the manuscript, who would be able to hold the data and respond to external requests for data access? If so, please provide their contact information (i.e., email address). Please also provide details on how you will ensure persistent or long-term data storage and availability.

**Response**: Thank you for this requirement. While the de-identified dataset is held securely by the study team, we have updated our Data Availability Statement to indicate that data requests may be directed to the Singapore General Hospital Research & Innovation Office (rio@sgh.com.sg). The Research Office serves as the institutional point of contact and will facilitate access requests to ensure long-term availability, even if individual authors are not contactable.

4. Please amend your list of authors on the manuscript to ensure that each author is linked to an affiliation. Authors’ affiliations should reflect the institution where the work was done (if authors moved subsequently, you can also list the new affiliation stating “current affiliation:….” as necessary).

**Response**: Thank you for your note. We amended the list of authors to ensure that each author was linked to an affiliation. The affiliations reflected the institutions where the work was carried out.

5. Please include captions for your Supporting Information files at the end of your manuscript, and update any in-text citations to match accordingly. Please see our Supporting Information guidelines for more information: [https://ddei5-0-ctp.trendmicro.com:443/wis/clicktime/v1/query?url=http%3a%2f%2fjournals.plos.org%2fplosone%2fs%2fsupporting%2dinformation&umid=FDD5C04E-3CB4-8206-8CB9-F3816F9BE5A2&auth=506d7fb60e41bcf4ca9a42eb3a951ef6efc5338b-0ff7d96b1f8830e49c1271cc9ace6f2b16733d16](https://ddei5-0-ctp.trendmicro.com/wis/clicktime/v1/query?url=http%3a%2f%2fjournals.plos.org%2fplosone%2fs%2fsupporting%2dinformation&umid=FDD5C04E-3CB4-8206-8CB9-F3816F9BE5A2&auth=506d7fb60e41bcf4ca9a42eb3a951ef6efc5338b-0ff7d96b1f8830e49c1271cc9ace6f2b16733d16).

**Response**: Thank you for this guidance. Captions for all Supporting Information files have now been added at the end of the manuscript, in line with the PLOS ONE guidelines. In-text citations have also been reviewed and updated to ensure consistency with the naming conventions (e.g., S1 Table, S2 Table).

6. Please remove all personal information, ensure that the data shared are in accordance with participant consent, and re-upload a fully anonymized data set.

Note: spreadsheet columns with personal information must be removed and not hidden as all hidden columns will appear in the published file.

Additional guidance on preparing raw data for publication can be found in our Data Policy ([https://ddei5-0-ctp.trendmicro.com:443/wis/clicktime/v1/query?url=https%3a%2f%2fjournals.plos.org%2fplosone%2fs%2fdata%2davailability%23loc%2dhuman%2dresearch%2dparticipant%2ddata%2dand%2dother%2dsensitive%2ddata&umid=FDD5C04E-3CB4-8206-8CB9-F3816F9BE5A2&auth=506d7fb60e41bcf4ca9a42eb3a951ef6efc5338b-fd881525eccd38bebb0e53376b7df9ffbc2653f0](https://ddei5-0-ctp.trendmicro.com/wis/clicktime/v1/query?url=https%3a%2f%2fjournals.plos.org%2fplosone%2fs%2fdata%2davailability%23loc%2dhuman%2dresearch%2dparticipant%2ddata%2dand%2dother%2dsensitive%2ddata&umid=FDD5C04E-3CB4-8206-8CB9-F3816F9BE5A2&auth=506d7fb60e41bcf4ca9a42eb3a951ef6efc5338b-fd881525eccd38bebb0e53376b7df9ffbc2653f0)) and in the following article: [https://ddei5-0-ctp.trendmicro.com:443/wis/clicktime/v1/query?url=http%3a%2f%2fwww.bmj.com%2fcontent%2f340%2fbmj.c181.long&umid=FDD5C04E-3CB4-8206-8CB9-F3816F9BE5A2&auth=506d7fb60e41bcf4ca9a42eb3a951ef6efc5338b-864fe7c617d005038eca69aa1438862e2259ce07](https://ddei5-0-ctp.trendmicro.com/wis/clicktime/v1/query?url=http%3a%2f%2ftrack.editorialmanager.com%2fCL0%2fhttp%3a%252F%252Fwww.bmj.com%252Fcontent%252F340%252Fbmj.c181.long%2f1%2f010f0198c1aa9ae5%2d72b763b3%2dae12%2d4657%2db757%2d5efd13e8916e%2d000000%2fus0FSqIJYgZq1OBMibdiEVyjcTnR7r7Lw1xYzTmQGLI%3d224&umid=FDD5C04E-3CB4-8206-8CB9-F3816F9BE5A2&auth=506d7fb60e41bcf4ca9a42eb3a951ef6efc5338b-b0742cc1b2be90b03be9e273292eb68a025778c6).

**Response**: We would like to clarify that no raw dataset containing personal information was submitted. Only de-identified, aggregate data are included in the manuscript tables. All information presented is fully anonymized and in line with participant consent.

7. If the reviewer comments include a recommendation to cite specific previously published works, please review and evaluate these publications to determine whether they are relevant and should be cited. There is no requirement to cite these works unless the editor has indicated otherwise.

**Response**: Thank you for the clarification. In this round of reviews, no specific publications were recommended for citation, and therefore no additional references were added.

Additional Editor Comments:

Manuscript ID: PONE-D-25-28987
Title: What do Older Adults Think of Youth Community Health Volunteers (YCHVs)? Perceptions and Experiences of Older Adults in a Youth-Led Post-Health Screening Advocacy Program in Singapore: A Qualitative Study
Editorial Decision: Major Revision
Editorial Comments to the Authors

Thank you for submitting your manuscript to PLOS ONE. Both reviewers agree that your study addresses an important and underexplored area in intergenerational and community health, particularly in the context of Singapore’s evolving approaches to chronic disease management. The reviewers find merit in your focus on the perspectives of older adults within a youth-led community health volunteer initiative. However, they have highlighted several areas that require substantive revision before the paper can be considered further.

In particular, reviewers identified the following key issues that must be addressed:

1. Clarity and Completeness of Program Description
Both reviewers noted insufficient detail on the Youth Community Health Volunteer (YCHV) program and the HealthStart intervention. Please expand your description to include:
• Youth CHVs’ age range, recruitment sources, training length, evaluation or competency checks, languages spoken, and whether their participation fulfills academic or community service requirements.
• Basic structure and duration of the HealthStart program (centre-based vs. home-based, individual vs. group delivery).
• Approximate number of YCHVs involved.
This level of detail is essential for readers unfamiliar with the Singaporean context and for ensuring transparency in program description.

**Response:** Thank you for the suggestions. We have expanded the description of the HealthStart program to include additional details on its structure, the role and training of YCHVs, and the nature of participant engagement.

Methods – Study Site (last portion)

*YCHVs, aged 15–35 years, were recruited from schools, volunteer portals, and youth networks, with no prior healthcare training required. All YCHVs completed a blended training programme with online modules and a one-day workshop on chronic disease management, SMART goal setting, motivational interviewing, and the Singapore healthcare system. Competency was assessed through quizzes and role-plays, and only those who passed were deployed. Each HealthStart team comprised one healthcare volunteer (doctor, nurse, or allied health professional) and four YCHVs, with each YCHV typically supporting two older adults. Across two programme cycles, 149 YCHVs were recruited, 138 trained, and 102 deployed (mean age 24 years; 74% female, mostly post-secondary students). YCHVs followed participants for three months with at least six in-person or virtual sessions, helping them understand their conditions, enrol with a primary care provider, use a health app (HealthHub or Healthy365), and set personalised SMART lifestyle goals under professional supervision.*

2. Methodological Transparency
Several methodological elements require clarification or addition:
• State explicitly the language of interviews (English, or otherwise), including procedures for transcription/translation where relevant.

**Response:** We have added information on the languages in which interviews were conducted, as well as the processes for transcription and translation. These details are now included in the Data Collection section, with additional reflections on how language and transcription were managed provided under Reflexivity.

Methods – Data Collection (last portion)

*Interviews were conducted in English or Mandarin, depending on participant preference. For Mandarin interviews, audio recordings were translated directly into English transcripts by bilingual co-authors fluent in both languages to ensure accuracy and consistency.*

Methods – Reflexivity (last portion)

*The authors recognize that direct translation from Mandarin to English may have introduced minor loss of nuance, which they mitigated through cross-checking by bilingual researchers.*

• Describe interviewer training and background.

**Response:** Thank you for the suggestion. We have added details on the training background of the interviewers. This information has been incorporated into the Data Collection section.

Methods – Data Collection

*Data were collected through one-to-one, semi-structured interviews with older adults conducted between November 2023 and January 2024. Interviews were conducted in a quiet room in community centers, healthcare facilities, or via telephone or Zoom (Zoom Video Communications, Inc., San Jose, California) by four co-authors (ASTK, JYRC, JJ, EWS), all affiliated with the Division of Population Health and Integrated Care (PHIC) at Singapore General Hospital. The interviewers were public health researchers or researchers-in-training, relatively new to qualitative research, and received guidance on the use of the interview guide to ensure consistency across interviews. Interviews were supervised by the qualitative lead (ASTK).*

• Specify the setting in which interviews were conducted (e.g., quiet room, community centre, home visits).

**Response:** Thank you for this comment. We have clarified that face-to-face interviews were conducted in quiet rooms at community centres or healthcare facilities, depending on participant preference and convenience. This detail has been added to the Data Collection section.

Methods – Data Collection

*Interviews were conducted in a quiet room in community centers, healthcare facilities, or via telephone or Zoom.*

• Include the sampling method and study setting in the abstract.

**Response:** The sampling method and setting have been added in the Abstract

Abstract- Methods

*We conducted one-to-one semi-structured interviews with 19 older adults who had participated in HealthStart, a YCHV-led post-health screening program. Purposive sampling was used to ensure representation across diverse demographic backgrounds and varying levels of program engagement. Interviews were carried out between November 2023 and January 2024 in community centers, healthcare facilities, or via telephone/Zoom, and explored participants’ experiences with the program, perceptions of YCHVs, and health-related outcomes. We analyzed data thematically using deductive and inductive coding.*

• Consider including your interview guide/questions in a supplementary table for transparency.

**Response:** We have appended the interview guide in Supplementary Table 1.

• Report your study in line with the Consolidated Criteria for Reporting Qualitative Research (COREQ) or similar framework to strengthen methodological rigor.

**Response**: Thank you for this valuable suggestion. We have now mapped our study against the COREQ checklist and confirmed that the manuscript addresses the majority of key items. To further strengthen methodological rigor, we have added clarifications in the Methods and Reflexivity sections regarding interviewer training and background, method of participant approach, non-participation, presence of non-participants, field notes, and transcript return. A completed COREQ checklist has also been submitted as Supporting Information (S2 Table).

Sampling and Recruitment

*Eligible participants were contacted by phone or text messages after screening and invited to participate. A few individuals declined participation, though the exact number was not systematically recorded.*

Data collection

*Only the interviewer and participant were present during each interview. Each interview lasted approximately 60 minutes, was scheduled at participants’ convenience, audio-recorded, and accompanied by field notes documenting contextual observations… Transcripts were not returned to participants for comment or correction.*

Reflexivity

*Participants were informed that interviewers were researchers from SGH and TriGen evaluating the HealthStart program.*

3. Presentation of Findings
The presentation of qualitative data requires refinement for readability and evidence support:
• Shorten long quotations for easier reading while retaining meaning.

**Response:** Thank you for the suggestion. The long quotations have been shortened for easy reading. Two such examples are listed below:

Subtheme 1.3:

*"[YCHV] is actually very good. She just said, need to exercise. At that time, I was quite fat, quite heavy, and she said to cut down on eating these [unhealthy] things. I listened to her… and lost a few kilograms... Previously, I would go and buy take-out for breakfast, and they were oily. Then, after I listened to her, I started eating oatmeal every day... whenever I cook dishes, I don’t use oil. Keep making soup… and pair it with rice. For vegetables... I use blanching to cook… And then, afterward, I told my daughter—I live with my daughter—'You need to eat these with me.’" (OA05)*

Subtheme 5.1:

*“[YCHV told me] there was a set of goals after I join HealthierSG (a national program linking residents to a primary care doctor and supporting them to set healthy lifestyle goals). I say, “where’s my goals? I got no goals”, then she say “oh yah, you look at the HealthHub (local health app)”, ... So, there’s actually goals set down there, but … maybe the doctor don’t have so much time, because there’s so many patients waiting… so they assume that we know how to go and see. But without (YCHV) coming in, nobody know that there’s a goal down there, you know.” (OA15)*

• Where only one quotation supports a point, consider providing an additional supporting quotation to increase evidence robustness.

**Response:** Thank you for the comments. The following quotes have been added at the respective lines:

*Line 155:*

*Another participant highlighted how he had not initially appreciated the seriousness of his newly diagnosed condition until YCHVs explained its implications:*

*“Interviewer03: For you, before [YCHV] came—because you have high cholesterol and such… did you feel that it was serious?*

*OA05: No ah… [But] the volunteers said “even though it is not that high yet but this is only the start, and you still don’t pay attention”, so I listened...”*

*Line 312*

*“[The program] did help to remind those patients, like us who went for the health screening [of healthier lifestyle habits]. Whether it did help or not, it is quite dependent on the individual.” (OA10)*

*Line 340*

*“[The program] did help to remind those patients, like us who went for the health screening [of healthier lifestyle habits]. Whether it did help or not, it is quite dependent on the individual.” (OA10)*

*Line 378- available excerpts beyond the one presented were brief and overlapping, and did not provide sufficient depth for inclusion. In this case, we synthesised these views in summary form rather than inserting multiple short fragments, to preserve clarity and analytic balance. We believe this approach better reflects the range of perspectives while avoiding over-representation of thin data.*

*Line 410-412- We have included an additional illustrative quotation (OA07) to strengthen this sub-theme. Other available quotes on this point were brief and did not provide sufficient depth for inclusion; therefore, we summarised those perspectives instead to preserve clarity and analytic balance.*

*“I mean to talk to old people is a bit difficult because they have lived such a long life ah… and they will not listen to people younger. … They are stubborn… So if (a health scare happens to their) surrounding friends ah, they saw it then they will feel scared then they want to do something to their health.” (OA07)*

• Add direct quotations in places where findings are reported in summary form (e.g., “one participant shared…”).

**Response:** Thank you for the comments. The following quotes have been added at the respective lines:

*Line 161 - The available quotation was brief and descriptive without providing additional depth beyond what was already summarized. In this case, we retained the summary to better reflect the analytic meaning while avoiding over-representing a thin excerpt.*

*Line 268*

*Interviewer03: Understand, understand. Do you feel that the results were serious?*

*R09: It was okay leh… When you’re older, all these blood pressure and blood sugar would be high. For instance, my blood pressure is around 130 or 140.*

*Line 272*

*“If I can’t work, I don’t have income mah. I have to go for doctor’s appointment, I still need to, (look out) for myself... Then I still have my mother whom I have to care for ah. Eighty plus years old... Her blood pressure when she measures, (the readings are) 170+, 180+, 160+... I am in pain all over, I don’t… I don’t have the…mood lah...” (OA18)*

• Insert quotations into long descriptive paragraphs to balance interpretation with participant voices.

**Response**: Thank you for this suggestion. We agree that including direct participant voices is important and have added additional illustrative quotations where appropriate. However, for the 2 longer paragraphs, the information was drawn from several participants whose comments were brief and overlapping. In these cases, we summarised the views rather than inserting shorter quotes, as this provided a clearer synthesis without over-representing thin excerpts. We believe this approach maintains balance between participants’ voices and analytic clarity.

• Review the terminology used for presenting themes (e.g., use “revealed” instead of “explored” to better reflect findings).

**Response:** Thank you for the suggestion. The verbs in the RESULT section have been changed accordingly.

• Where possible, analyze differences between participants who engaged with YCHVs for fewer vs. more sessions, as this may yield practical insights.

**Response**: Thank you for this insightful suggestion. We agree that differences in perceptions based on the number of YCHV engagements would be an important area to explore. However, this level of comparison was not within the scope of the present qualitative paper, which focused on understanding the breadth of older adults’ experiences and perspectives. A separate mixed-methods evaluation of the HealthStart programme has been conducted, which includes analysis of engagement intensity and its associations with health outcomes. We have noted this in the Discussion as an area for further research and cross-reference the broader evaluation work.

*Discussion*

*Finally, while this study focused on capturing the breadth of older adults’ experiences, it did not compare perceptions by number of YCHV engagements. Such analyses were beyond the scope of this qualitative paper but are being addressed in a separate mixed-methods evaluation of the HealthStart programme.*

4. Contextualization and Discussion
To aid international readers:
• Provide a brief explanation of Singapore’s healthcare system (e.g., availability of universal health coverage and its implications).

**Response:** Thank you for this suggestion. We agree that providing context on Singapore’s healthcare system would be helpful for international readers. We have therefore added a brief description in the Study Site section

Methods - Study Site

*The program was embedded in Singapore’s public healthcare system, where government subsidies reduce out-of-pocket costs for primary and specialist care. Universal catastrophic coverage is provided through MediShield Life, Singapore’s national health insurance scheme, complemented by compulsory medical savings (MediSave) and a safety net fund (MediFund). This mixed financing model differs from countries without government-sponsored coverage, where affordability may be a significant barrier to follow-up.*

• Clarify Singapore-specific terminology used in participant quotes.

**Response:** Thank you, we have made the necessary edits. Connector words have been added for the quote in Line 277-286 for clarity.

Sub-Theme 2.2

*“Sometimes need [a] third party to talk [to the older adults], then [they] will listen ah. [For]some people, when [their] wives or husbands talk, [they] don’t want to listen, [but] when third party talks ah, then they listen.” (OA06)*

• Avoid repeating themes in the Discussion without additional interpretation.

**Response:** The 5 themes have been removed from the Discussion.

• Refine the limitations section: your sample size is adequate for qualitative inquiry, but limitations relate more to representation and mode of data collection (e.g., face-to-face vs. non-face-to-face).

**Response:** Thank you for this observation. We have clarified in the Limitations section that while most interviews were conducted face-to-face, three were conducted by telephone and one via Zoom. We acknowledge that variation in interview mode may have influenced rapport and the depth of responses, although the impact was likely minimal given that the majority of interviews were conducted in person.

Discussion - last paragraph

*Although three interviews were conducted by telephone and one via Zoom, the majority were face-to-face, minimising the potential impact of mode on rapport and depth of responses.*

• Consider discussing whether a minimum threshold of YCHV engagements is necessary to generate measurable benefit.

**Response**: Thank you for this insightful suggestion. We agree that differences in perceptions and benefits gained based on the number of YCHV engagements would be an important area to explore. However, this level of comparison was not within the scope of the present qualitative paper, which focused on understanding the breadth of older adults’ experiences and perspectives. A separate mixed-methods evaluation of the HealthStart programme has been conducted, which includes analysis of engagement intensity and its associations with health outcomes. We have noted this in the Discussion as an area for further research and cross-reference the broader evaluation work.

*Discussion*

*Finally, while this study focused on capturing the breadth of older adults’ experiences, it did not compare perceptions by number of YCHV engagements. Such analyses were beyond the scope of this qualitative paper but are being addressed in a separate mixed-methods evaluation of the HealthStart programme.*

5. Language and Style
• Correct specific textual issues noted by Reviewer 1 (e.g., line 184 redundancy, line 224 phrasing, “enlightening” on line 332, and formatting of Conclusion).

**Response:** Thank you, we have made the necessary edits.

• Adjust manuscript title for conciseness as suggested by Reviewer 2: Perceptions and Experiences of Older Adults in a Youth-Led Post-Health Screening Advocacy Program in Singapore: A Qualitative Study.

**Response:** We have refined the title and retained the keyword ‘Youth Community Health Volunteer’

Title

*Perceptions and Experiences of Older Adults in a Youth Community Health Volunteer–Led Health Coaching Programme in Singapore: A Qualitative Study*

Decision Summary
Your manuscript makes a valuable contribution, but it currently requires major revision to meet PLOS ONE’s standards for methodological transparency, qualitative rigor, and accessibility to an international readership. Please carefully address all reviewer concerns point by point in your rebuttal letter and highlight revisions in your manuscript.
We look forward to receiving your revised submission.
Sincerely,
Prof. Morufu Olalekan Raimi
Academic Editor
PLOS ONE


[Note: HTML markup is below. Please do not edit.]

Reviewers' comments:

Reviewer's Responses to Questions

**Comments to the Author**

1. Is the manuscript technically sound, and do the data support the conclusions?

The manuscript must describe a technically sound piece of scientific research with data that supports the conclusions. Experiments must have been conducted rigorously, with appropriate controls, replication, and sample sizes. The conclusions must be drawn appropriately based on the data presented.

Reviewer #1: Yes

Reviewer #2: Yes

2. Has the statistical analysis been performed appropriately and rigorously?

Reviewer #1: Yes

Reviewer #2: Yes

3. Have the authors made all data underlying the findings in their manuscript fully available?

The [PLOS Data policy](https://ddei5-0-ctp.trendmicro.com/wis/clicktime/v1/query?url=http%3a%2f%2ftrack.editorialmanager.com%2fCL0%2fhttp%3a%252F%252Fwww.plosone.org%252Fstatic%252Fpolicies.action%2523sharing%2f1%2f010f0198c1aa9ae5%2d72b763b3%2dae12%2d4657%2db757%2d5efd13e8916e%2d000000%2fy8nmu2tiTJ3zKN9LKa9GGx7V2BG9MO8HWRrTuEwrjxI%3d224&umid=FDD5C04E-3CB4-8206-8CB9-F3816F9BE5A2&auth=506d7fb60e41bcf4ca9a42eb3a951ef6efc5338b-d648ce4fc8bc4db55ba3d939f066faf4b83230db) requires authors to make all data underlying the findings described in their manuscript fully available without restriction, with rare exception (please refer to the Data Availability Statement in the manuscript PDF file). The data should be provided as part of the manuscript or its supporting information, or deposited to a public repository. For example, in addition to summary statistics, the data points behind means, medians and variance measures should be available. If there are restrictions on publicly sharing data—e.g. participant privacy or use of data from a third party—those must be specified.

Reviewer #1: Yes

Reviewer #2: No

4. Is the manuscript presented in an intelligible fashion and written in standard English?

PLOS ONE does not copyedit accepted manuscripts, so the language in submitted articles must be clear, correct, and unambiguous. Any typographical or grammatical errors should be corrected at revision, so please note any specific errors here.

Reviewer #1: Yes

Reviewer #2: Yes

5. Review Comments to the Author

Please use the space provided to explain your answers to the questions above. You may also include additional comments for the author, including concerns about dual publication, research ethics, or publication ethics. (Please upload your review as an attachment if it exceeds 20,000 characters)

Reviewer #1: This is an interesting and important qualitative study of an intergenerational intervention for chronic disease management.
There are a few things that would make the paper stronger:

1. While the authors cite pre-publications detailing the Healthstart program and their mixed methods evaluation, this paper would benefit from more detail on the youth CHV program - specifically: how old are the youth, how long is their training, from where are they recruited, does this provide class credit or community service requirement fulfillment, how many youth CHVs are there, what languages do they speak, etc.

**Response:** Thank you for the suggestion. We have expanded the description of the HealthStart program to include additional details on its structure, the role and training of YCHVs, and the nature of participant engagement.

Methods – Study Site (last portion)

*YCHVs, aged 15–35 years, were recruited from schools, volunteer portals, and youth networks, with no prior healthcare training required. All YCHVs completed a blended training programme with online modules and a one-day workshop on chronic disease management, SMART goal setting, motivational interviewing, and the Singapore healthcare system. Competency was assessed through quizzes and role-plays, and only those who passed were deployed. Each HealthStart team comprised one healthcare volunteer (doctor, nurse, or allied health professional) and four YCHVs, with each YCHV typically supporting two older adults. YCHVs followed participants for three months with at least six in-person or virtual sessions, helping them understand their conditions, enrol with a primary care provider, use a health app (HealthHub or Healthy365), and set personalised SMART lifestyle goals under professional supervision. Across two programme cycles, 149 YCHVs were recruited, 138 trained, and 102 deployed (mean age 24 years; 74% female, mostly post-secondary students).*

2. Also in the methods, include that interviews were conducted in English, which is one of the official languages of Singapore. Not all readers will be familiar with this.

**Response:** Thank you for the feedback. We have added information on the languages in which interviews were conducted, as well as the processes for transcription and translation. These details are now included in the Data Collection section, with additional reflections on how language and transcription were managed provided under Reflexivity.

Methods – Data Collection (last portion)

*Interviews were conducted in English or Mandarin, depending on participant preference. For Mandarin interviews, audio recordings were translated directly into English transcripts by bilingual co-authors fluent in both languages to ensure accuracy and consistency.*

Methods – Reflexivity (last portion)

*The authors recognize that direct translation from Mandarin to English may have introduced minor loss of nuance, which they mitigated through cross-checking by bilingual researchers.*

3. It may also be helpful to the reader to understand whether Singapore has government-sponsored, universal healthcare - as this is a barrier that some face in other countries (e.g. US).

**Response:** Thank you for this suggestion. We agree that providing context on Singapore’s healthcare system would be helpful for international readers. We have therefore added a brief description in the Study Site section

Methods - Study Site

*The program was embedded in Singapore’s public healthcare system, where government subsidies reduce out-of-pocket costs for primary and specialist care. Universal catastrophic coverage is provided through MediShield Life, Singapore’s national health insurance scheme, complemented by compulsory medical savings (MediSave) and a safety net fund (MediFund). This mixed financing model differs from countries without government-sponsored coverage, where affordability may be a significant barrier to follow-up.*

4. It would also be useful to include the interview questions in a table.

**Response:** We have appended the interview guide in Supplementary Table 1.

5. Specific line item edits:
Line 184: “such as “reducing reduced their consumption of sugar-sweetened”
Line 224: “to foster of trust and accountability”
Line 277-286 - for the non-resident of Singapore, this quote would benefit from a little more explanation of the terminology used.
Line 332 - perhaps a different word than “enlightening” - - this doesn’t seem to fit.
Line 518: CONCLUSION

**Response:** Thank you, we have made the necessary edits. Connector words have been added for the quote in Line 277-286 for clarity.

Sub-Theme 2.2

*“Sometimes need [a] third party to talk [to the older adults], then [they] will listen ah. [For]some people, when [their] wives or husbands talk, [they] don’t want to listen, [but] when third party talks ah, then they listen.” (OA06)*

Reviewer #2: Review comments:
Thanks for giving me this opportunity to review this manuscript. This study explored older adults’ experiences and perceptions of a YCHV-led health advocacy program following community health screening in Singapore.
Below are my comments for your consideration and improvement:

Title
1. Suggest making the manuscript title more concise: Perceptions and Experiences of Older Adults in a Youth-Led Post-Health Screening Advocacy Program in Singapore: A Qualitative Study

**Response:** Thank you for the feedback. We have refined the title and retained the keyword ‘Youth Community Health Volunteer’

Title

*Perceptions and Experiences of Older Adults in a Youth Community Health Volunteer–Led Health Coaching Programme in Singapore: A Qualitative Study*

Abstract
2. Please add your sampling method and setting.

**Response:** The sampling method and setting have been added in the Abstract

Abstract- Methods

*We conducted one-to-one semi-structured interviews with 19 older adults who had participated in HealthStart, a YCHV-led post-health screening program. Purposive sampling was used to ensure representation across diverse demographic backgrounds and varying levels of program engagement. Interviews were carried out between November 2023 and January 2024 in community centers, healthcare facilities, or via telephone/Zoom, and explored participants’ experiences with the program, perceptions of YCHVs, and health-related outcomes. We analyzed data thematically using deductive and inductive coding.*

3. The first sentence in your conclusion should be shifted to the “results” section.

**Response**: Thank you for the suggestion. After careful consideration, we have retained the first sentence in the Conclusion section. We feel it functions best as a framing statement that synthesizes the key findings and sets up the subsequent implications, rather than as a results-oriented statement. We believe this positioning maintains clarity and flow, while the Results section already presents the relevant findings in detail.

Introduction

4. To improve the quality of reporting qualitative data, I will suggest that authors consider using “The consolidated criteria for reporting qualitative research”.

**Response**: Thank you for this valuable suggestion. We have now mapped our study against the COREQ checklist and confirmed that the manuscript addresses the majority of key items. To further strengthen methodological rigor, we have added clarifications in the Methods and Reflexivity sections regarding interviewer training and background, method of participant approach, non-participation, presence of non-participants, field notes, and transcript return. A completed COREQ checklist has also been submitted as Supporting Information (S2 Table).

Sampling and Recruitment

*Eligible participants were contacted by phone or text messages after screening and invited to participate. A few individuals declined participation, though the exact number was not systematically recorded.*

Data collection

*Only the interviewer and participant were present during each interview. Each interview lasted approximately 60 minutes, was scheduled at participants’ convenience, audio-recorded, and accompanied by field notes documenting contextual observations… Transcripts were not returned to participants for comment or correction.*

Reflexivity

*Participants were informed that interviewers were researchers from SGH and TriGen evaluating the HealthStart program.*

Method
Under “study site”,

5. Page 2, please provide some information on the characteristics of the youth volunteers, e.g., age range, training background, and how many days of training they received? any competency check post-training?

**Response:** Thank you for the suggestion. We have expanded the description of the HealthStart program to include the characteristics of the youth volunteers.

Methods – Study Site (last portion)

*YCHVs, aged 15–35 years, were recruited from schools, volunteer portals, and youth networks, with no prior healthcare training required. All YCHVs completed a blended training programme with online modules and a one-day workshop on chronic disease management, SMART goal setting, motivational interviewing, and the Singapore healthcare system. Competency was assessed through quizzes and role-plays, and only those who passed were deployed. Each HealthStart team comprised one healthcare volunteer (doctor, nurse, or allied health professional) and four YCHVs, with each YCHV typically supporting two older adults. Across two programme cycles, 149 YCHVs were recruited, 138 trained, and 102 deployed (mean age 24 years; 74% female, mostly post-secondary students). YCHVs followed participants for three months with at least six in-person or virtual sessions, helping them understand their conditions, enrol with a primary care provider, use a health app (HealthHub or Healthy365), and set personalised SMART lifestyle goals under professional supervision.*

6. Page 3, can you provide a short statement on the HealthStart program? how long is the program? is it centre-based? one-to-one or group?
Under “data collection”

**Response:** Thank you for the suggestion. We have expanded the description of the HealthStart program to include additional details on its structure.

Methods – Study Site (last portion)

*YCHVs, aged 15–35 years, were recruited from schools, volunteer portals, and youth networks, with no prior healthcare training required. All YCHVs completed a blended training programme with online modules and a one-day workshop on chronic disease management, SMART goal setting, motivational interviewing, and the Singapore healthcare system. Competency was assessed through quizzes and role-plays, and only those who passed were deployed. Each HealthStart team comprised one healthcare volunteer (doctor, nurse, or allied health professional) and four YCHVs, with each YCHV typically supporting two older adults. Across two programme cycles, 149 YCHVs were recruited, 138 trained, and 102 deployed (mean age 24 years; 74% female, mostly post-secondary students). YCHVs followed participants for three months with at least six in-person or virtual sessions, helping them understand their conditions, enrol with a primary care provider, use a health app (HealthHub or Healthy365), and set personalised SMART lifestyle goals under professional supervision.*

7. Page 4, where did you conduct the interviews face-to-face? in a quiet room?

**Response:** Thank you for this comment. We have clarified that face-to-face interviews were conducted in quiet rooms at community centres or healthcare facilities, depending on participant preference and convenience. This detail has been added to the Data Collection section.

Methods – Data Collection

*Interviews were conducted in a quiet room in community centers, healthcare facilities, via telephone, or Zoom.*

8. What language did you use? Were all interviews conducted in English? If not, please describe how you recorded, transcribed, and translated.

**Response:** We have added information on the languages in which interviews were conducted, as well as the processes for transcription and translation. These details are now included in the Data Collection section, with additional reflections on how language and transcription were managed provided under Reflexivity.

Methods – Data Collection (last portion)

*Interviews were conducted in English or Mandarin, depending on participant preference. For Mandarin interviews, audio recordings were translated directly into English transcripts by bilingual co-authors fluent in both languages to ensure accuracy and consistency.*

Methods – Reflexivity (last portion)

*The authors recognize that direct translation from Mandarin to English may have introduced minor loss of nuance, which they mitigated through cross-checking by bilingual researchers.*

9. Please state the training background of the interviewers.

**Response:** Thank you for the suggestion. We have added details on the training background of the interviewers. This information has been incorporated into the Data Collection section.

Methods – Data Collection

*Data were collected through one-to-one, semi-structured interviews with older adults conducted between November 2023 and January 2024. Interviews were conducted in a quiet room in community centers, healthcare facilities, via telephone or Zoom (Zoom Video Communications, Inc., San Jose, California) by four co-authors (ASTK, JYRC, JJ, EWS), all affiliated with the Division of Population Health and Integrated Care (PHIC) at Singapore General Hospital. The interviewers were public health researchers or researchers-in-training, relatively new to qualitative research, and received guidance on the use of the interview guide to ensure consistency across interviews. Interviews were supervised by the qualitative lead (ASTK).*

RESULTS

10. As shown in Table 1, any differences in their perceptions between a smaller number of YCHV engagements and a higher number of YCHV engagements?

**Response**: Thank you for this insightful suggestion. We agree that differences in perceptions based on the number of YCHV engagements would be an important area to explore. However, this level of comparison was not within the scope of the present qualitative paper, which focused on understanding the breadth of older adults’ experiences and perspectives. A separate mixed-methods evaluation of the HealthStart programme has been conducted, which includes analysis of engagement intensity and its associations with health outcomes. We have noted this in the Discussion as an area for further research and cross-reference the broader evaluation work.

*Discussion*

*Finally, while this study focused on capturing the breadth of older adults’ experiences, it did not compare perceptions by number of YCHV engagements. Such analyses were beyond the scope of this qualitative paper but are being addressed in a separate mixed-methods evaluation of the HealthStart programme.*

11. When presenting your qualitative data, please consider shortening long quotes from just one participant to easy reading, e.g., lines 193-202; 277-286; 384-392;

**Response:** Thank you for the suggestion. The 3 quotes have been shortened for easy reading.

12. Please consider having at least two quotes (with participants’ codes) will increase the level of evidence in a qualitative study. E.g., line 155, line 312, 340, 378, 410-412;

**Response:** Thank you for the comments. The following quotes have been added at the respective lines:

*Line 155:*

*Another participant highlighted how he had not initially appreciated the seriousness of his newly diagnosed condition until YCHVs explained its implications:*

*“Interviewer03: For you, before [YCHV] came—because you have high cholesterol and such… did you feel that it was serious?*

*OA05: No ah… [But] the volunteers said “even though it is not that high yet but this is only the start, and you still don’t pay attention”, so I listened...”*

*Line 312*

*“[The program] did help to remind those patients, like us who went for the health screening [of healthier lifestyle habits]. Whether it did help or not, it is quite dependent on the individual.” (OA10)*

*Line 340*

*“[The program] did help to remind those patients, like us who went for the health screening [of healthier lifestyle habits]. Whether it did help or not, it is quite dependent on the individual.” (OA10)*

*Line 378- available excerpts beyond the one presented were brief and overlapping, and did not provide sufficient depth for inclusion. In this case, we synthesised these views in summary form rather than inserting multiple short fragments, to preserve clarity and analytic balance. We believe this approach better reflects the range of perspectives while avoiding over-representation of thin data.*

*Line 410-412- We have included an additional illustrative quotation (OA07) to strengthen this sub-theme. Other available quotes on this point were brief and did not provide sufficient depth for inclusion; therefore, we summarised those perspectives instead to preserve clarity and analytic balance.*

*“I mean to talk to old people is a bit difficult because they have lived such a long life ah… and they will not listen to people younger. … They are stubborn… So if (a health scare happens to their) surrounding friends ah, they saw it then they will feel scared then they want to do something to their health.” (OA07)*

13. When indicating “one participant shared …”, please add the original quote. E.g., line 161, 268, 272,

**Response:** Thank you for the comments. The following quotes have been added at the respective lines:

*Line 161 - The available quotation was brief and descriptive without providing additional depth beyond what was already summarized. In this case, we retained the summary to better reflect the analytic meaning while avoiding over-representing a thin excerpt.*

*Line 268*

*Interviewer03: Understand, understand. Do you feel that the results were serious?*

*R09: It was okay leh… When you’re older, all these blood pressure and blood sugar would be high. For instance, my blood pressure is around 130 or 140.*

*Line 272*

*“If I can’t work, I don’t have income mah. I have to go for doctor’s appointment, I still need to, (look out) for myself... Then I still have my mother whom I have to care for ah. Eighty plus years old... Her blood pressure when she measures, (the readings are) 170+, 180+, 160+... I am in pain all over, I don’t… I don’t have the…mood lah...” (OA18)*

14. Please consider adding quotes in your long paragraphs under different sub-themes. E.g., between lines180-190; 208-221;

**Response**: Thank you for this suggestion. We agree that including direct participant voices is important and have added additional illustrative quotations where appropriate. However, for the 2 longer paragraphs, the information was drawn from several participants whose comments were brief and overlapping. In these cases, we summarised the views rather than inserting shorter quotes, as this provided a clearer synthesis without over-representing thin excerpts. We believe this approach maintains balance between participants’ voices and analytic clarity.

15. Please change the verb “explored” to “revealed” when presenting themes/sub-themes in your RESULT section. e.g., line 261, 320, 368

**Response:** Thank you for the suggestion. The verbs in the RESULT section have been changed accordingly.

DISCUSSION
16. Lines 419-422: You don't have to repeat the 5 themes here.

**Response:** The 5 themes have been removed.

17. Line 511: As part of your study limitation, your sample size is sufficient for such a qualitative study, rather, the representation of the samples.

**Response:** Thank you for this observation. We have clarified in the Limitations section that while most interviews were conducted face-to-face, three were conducted by telephone and one via Zoom. We acknowledge that variation in interview mode may have influenced rapport and the depth of responses, although the impact was likely minimal given that the majority of interviews were conducted in person.

Discussion - last paragraph

*Although three interviews were conducted by telephone and one via Zoom, the majority were face-to-face, minimising the potential impact of mode on rapport and depth of responses.*

18. Under your study limitations, were interviews conducted non-f2f your limitations?

**Response:** Thank you for this observation. We have clarified in the Limitations section that while most interviews were conducted face-to-face, three were conducted by telephone and one via Zoom. We acknowledge that variation in interview mode may have influenced rapport and the depth of responses, although the impact was likely minimal given that the majority of interviews were conducted in person.

Discussion - last paragraph

*Although three interviews were conducted by telephone and one via Zoom, the majority were face-to-face, minimising the potential impact of mode on rapport and depth of responses.*

19. It will be good to have a discussion on the differences in perception between those who received 1-3 YCHV sessions and 4-7 sessions, which may inform future practices, like setting a minimum number of YCHV engagement sessions to be more beneficial.

**Response**: Thank you for this insightful suggestion. We agree that differences in perceptions based on the number of YCHV engagements would be an important area to explore. However, this level of comparison was not within the scope of the present qualitative paper, which focused on understanding the breadth of older adults’ experiences and perspectives. A separate mixed-methods evaluation of the HealthStart programme has been conducted, which includes analysis of engagement intensity and its associations with health outcomes. We have noted this in the Discussion as an area for further research and cross-reference the broader evaluation work.

*Discussion*

*Finally, while this study focused on capturing the breadth of older adults’ experiences, it did not compare perceptions by number of YCHV engagements. Such analyses were beyond the scope of this qualitative paper but are being addressed in a separate mixed-methods evaluation of the HealthStart programme.*

6. PLOS authors have the option to publish the peer review history of their article ([what does this mean?](https://ddei5-0-ctp.trendmicro.com/wis/clicktime/v1/query?url=https%3a%2f%2ftrack.editorialmanager.com%2fCL0%2fhttps%3a%252F%252Fjournals.plos.org%252Fplosone%252Fs%252Feditorial%2dand%2dpeer%2dreview%2dprocess%2523loc%2dpeer%2dreview%2dhistory%2f1%2f010f0198c1aa9ae5%2d72b763b3%2dae12%2d4657%2db757%2d5efd13e8916e%2d000000%2fKxCTW0ZsA6cczpSidLZTGczgSrekdqRC%5fOMUJASEv80%3d224&umid=FDD5C04E-3CB4-8206-8CB9-F3816F9BE5A2&auth=506d7fb60e41bcf4ca9a42eb3a951ef6efc5338b-5c0926c34418e19b5cd42d03279b7ffc0cc087ce)). If published, this will include your full peer review and any attached files.

If you choose “no”, your identity will remain anonymous but your review may still be made public.

**Do you want your identity to be public for this peer review?** For information about this choice, including consent withdrawal, please see our [Privacy Policy](https://ddei5-0-ctp.trendmicro.com/wis/clicktime/v1/query?url=https%3a%2f%2ftrack.editorialmanager.com%2fCL0%2fhttps%3a%252F%252Fwww.plos.org%252Fprivacy%2dpolicy%2f1%2f010f0198c1aa9ae5%2d72b763b3%2dae12%2d4657%2db757%2d5efd13e8916e%2d000000%2ftCd73T3vtjtMmC9krYmOhSzNHGviP%5f8YWPfmlKsiU4Y%3d224&umid=FDD5C04E-3CB4-8206-8CB9-F3816F9BE5A2&auth=506d7fb60e41bcf4ca9a42eb3a951ef6efc5338b-4809aa6ac2aa9d86cc9673fcb94cfb75ca156268).

Reviewer #1: **Yes:** Renee Cadzow

Reviewer #2: **Yes:** Tianma Xu
